# Day and Night, Night and Day: Associations of Sleep and Activity Variability and Mortality

**DOI:** 10.1093/geroni/igab046.1460

**Published:** 2021-12-17

**Authors:** Jade Benson, Elena Jauregui, Diane Lauderdale

**Affiliations:** 1 University of Chicago, Chicago, Illinois, United States; 2 The University of Chicago, Chicago, Illinois, United States

## Abstract

Self-reported sleep duration has been repeatedly found to predict mortality. Actigraphy has recently been added to population-based studies to provide more accurate sleep measures. Actigraphy sleep duration has not consistently predicted mortality, but actigraphy measures of sleep disruption measures are generally found to be predictive of mortality for older adults. A few studies have more fully used actigraphy data and constructed variables to summarize 24-hour activity patterns, which have also predicted mortality. In this study, we use a nationally representative study of Americans aged 61 – 91 to examine the associations between mortality and actigraphy-derived measures of variability, for both sleep and 24-hour activity patterns. We use 72-hour wrist actigraphy data from a substudy of the 2010/11 round of the National Social Life, Health and Aging Project (NSHAP) linked to the National Death Index (NDI) to establish 5-year mortality. Sleep variability was represented by sleep fragmentation and the standard deviation of wake and bed times. Intraday variability and between day (interday) variability described the 24-hour activity patterns. Cox proportional hazards models were adjusted for sociodemographic confounders and average daytime activity. In general, more variability was associated with increased death hazard for all measures. Fragmentation (HR: 1.04, 95% CI: [1.01, 1.07], p = 0.01), standard deviation of bedtimes (HR: 1.16, 95% CI: [1.02, 1.31], p = 0.02), and intraday variability (HR: 1.19, 95% CI: [0.98, 1.43], p = 0.07) showed the strongest associations. This study suggests that both consistent sleep and 24-hour activity patterns are associated with better prospective health.

